# New insights to diversity and enzyme–substrate interactions of fungal glucuronoyl esterases

**DOI:** 10.1007/s00253-023-12575-4

**Published:** 2023-05-31

**Authors:** Jane Wittrup Agger, Michael Schmidt Madsen, Line Korte Martinsen, Pedro Alves Martins, Kristian Barrett, Anne S. Meyer

**Affiliations:** Department of Biotechnology and Biomedicine, Technical University of Denmark, Søltofts Plads 224, 2800 Kongens Lyngby, Denmark

**Keywords:** Glucuronoyl esterases, Lignin-carbohydrate complexes, Lignin, CUPP, Molecular docking

## Abstract

**Abstract:**

Glucuronoyl esterases (GEs) (EC 3.1.1.117) catalyze the cleavage of ester-linked lignin-carbohydrate complexes that has high impact on the plant cell wall integrity. The GEs are among the very few known types of hydrolytic enzymes that act at the interface of lignin, or which may potentially interact with lignin itself. In this review, we provide the latest update of the current knowledge on GEs with a special focus on the fungal variants. In addition, we have established the phylogenetic relationship between all GEs and this reveals that the fungal enzymes largely fall into one major branch, together with only a minor subset of bacterial enzymes. About 22% of the fungal proteins carry an additional domain, which is almost exclusively a CBM1 binding domain. We address how GEs may interact with the lignin-side of their substrate by molecular docking experiments based on the known structure of the *Cerrena unicolor* GE (*Cu*GE). The docking studies indicate that there are no direct interactions between the enzyme and the lignin polymer, that the lignin-moiety is facing away from the protein surface and that an elongated carbon-chain between the ester-linkage and the first phenyl of lignin is preferable. Much basic research on these enzymes has been done over the past 15 years, but the next big step forward for these enzymes is connected to application and how these enzymes can facilitate the use of lignocellulose as a renewable resource.

**Key points:**

- *Fungal GEs are closely related and are sometimes linked to a binding module*
- *Molecular docking suggests good accommodation of lignin-like substructures*
- *GEs could be among the first expressed enzymes during fungal growth on biomass.*

**Supplementary Information:**

The online version contains supplementary material available at 10.1007/s00253-023-12575-4.

## Introduction

Glucuronoyl esterases (GEs) are presently the only type of enzymes known to attack and cleave lignin-carbohydrate complexes (LCCs). LCCs are covalent linkages between lignin and polysaccharides (primarily hemicellulose) in lignocellulose and they serve a fundamental role of making intermolecular connection points between the polymeric structures of plant cell walls. Exactly because these linkages represent key anchoring points in lignocellulose, the potential roles of GEs in future applications of lignocellulose are remarkable.

GEs belong to the carbohydrate esterase family 15 (CE15) in the Carbohydrate Active Enzymes database (http://www.cazy.org) (Drula et al. 2022). They were introduced in the CAZy collection in 2007 after their discovery in the secretome of *Schizophyllum commune* (Špániková and Biely 2006)*.* At that time, the activity of these enzymes was unknown, but the major hypothesis was that they catalyze the hydrolytic cleavage of ester-linked LCCs after observing hydrolysis of the methyl-ester of 4-*O*-methyl-d-glucuronic acid. This hypothesis has been the main driver for researchers engaged in studies of glucuronoyl esterases ever since, and indeed the evidence points toward this as a highly plausible biological function (Arnling Bååth et al. 2016, 2018; Mosbech et al. 2018). Due to the substantial evidence of the enzyme activity, GEs were recently assigned a new EC-number as EC.3.1.1.117; (4-*O*-methyl)-d-glucuronate-lignin-esterase.

Today, a variety of characterized CE15 proteins have been reported of both fungal (Ďuranová et al. 2009b; Pokkuluri et al. 2011; Charavgi et al. 2013; Katsimpouras et al. 2014; d’Errico et al. 2015; Mosbech et al. 2018, 2019; Dilokpimol et al. 2018; Tang et al. 2019) and bacterial origin (De Santi et al. 2016; Arnling Bååth et al. 2016, 2018; Baath et al. 2019; Krska et al. 2021). This review focuses on the occurrence and characteristics of fungal glucuronoyl esterases, and over the years, important reviews have been published, focusing on the early studies (Biely 2016; Monrad et al. 2018) and latest on the bacterial glucuronoyl esterases (Larsbrink and Lo Leggio 2023). At the time of this review, the online CAZy database contains 22 characterized CE15s with bacterial and fungal CE15s constituting exactly half each. However, the literature search conducted in connection with this review found 42 characterized CE15s (Table S1). More than two-thirds of these are fungal CE15s, and *Pichia pastoris* (*Komagataella phaffii*) was used as expression host in most studies. The majority of these enzymes are characterized on model substrates like benzyl- and methyl-glucuronates, whereas only a handful have been studied on authentic biomass or biomass fractions. All enzymes function at pH 5–7, between 25 and 50 °C and about half of the studied fungal proteins carry a CBM1. There is a clear tendency that K_M_ is lower on natural substrates or model substrate that closely mimic natural substrate compared to simple models (Table S1). Eight different CE15s, both fungal and bacterial, have been crystallized for structural studies, and three of these studies include complexes where the enzyme is bound to a relevant ligand (Charavgi et al. 2013; Mazurkewich et al. 2019; Ernst et al. 2020).

## The substrate

**Fig. 1:**
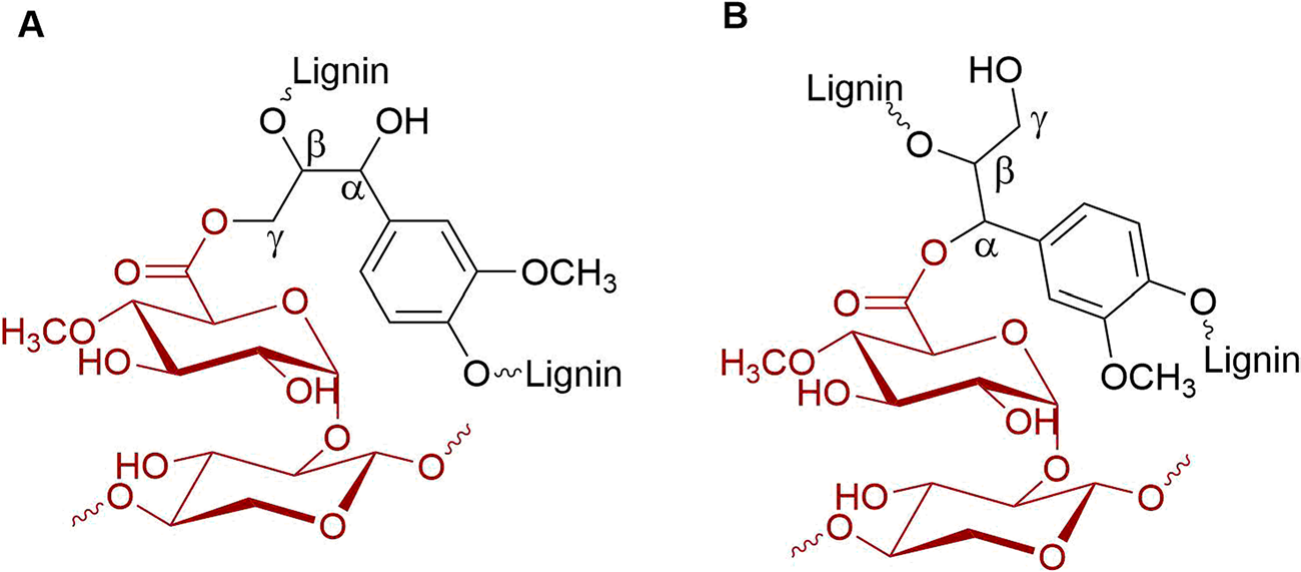
Structural representation of ester-linked LCCs between α-1,2-linked 4-O-methyl-d-glucuronoyls on xylan (marked in red) and the gamma-positioned hydroxyl (**A**) and the alpha-positioned hydroxyl (**B**) on lignin

LCCs exist in a number of different chemical conformations, but in the context of GE action the ester-linked LCCs are of interest. Most evidence point toward the direction that the ester-linked LCC exists between the carboxylic moiety of α-1,2-linked 4-*O*-methyl-d-glucuronoyl substitutions on glucuronoxylan and the γ-positioned hydroxyls on lignin (Fig. 1A) (Balakshin et al. 2011; Giummarella et al. 2016, 2019; Giummarella and Lawoko 2016). However, the debate about whether the ester also exists as the corresponding α-positioned ester continues (Fig. 1B). It is challenging to provide sufficient evidence for the existence of either of the two ester-forms due to highly overlapping regions in lignin-NMR spectra and low concentrations of the LCCs. However, new results from NMR experiments of LCC-enriched lignin fractions from spruce show the occurrence of both the gamma- and the benzyl-ester (where the latter corresponds to the alpha-ester) (Sapouna and Lawoko 2021), and suggest that both forms may be present in biomass. Indeed, the alpha-ester may be the initial form by which the ester is formed in the cell wall, whereas the gamma-ester occurs as a result of ester-migration to the resulting and resting gamma-position (Li and Helm 1995).

The composition of the ester-linked LCC entails that they predominantly exist in plants that contain glucuronoxylan, i.e., notably hardwoods. Despite the relatively well-defined ester bond in terms of alcohol and acid donors, the surrounding molecular construction of both the heterogeneous glucuronoxylan and the largely insoluble lignin adds a high level of complexity to the overall water-insoluble substrate. The ester-linkage itself is chemically prone to hydrolysis at both alkaline and acidic pH; typical process conditions during conventional pretreatment strategies. Consequently, ester-LCCs are often chemically hydrolyzed during intensive lignocellulose processing.

However, lignocellulose processing is facing a new era, which is often referred to as “Lignin-First” (Abu-Omar et al. 2021). The Lignin-First approach and other similar research directions emphasize the extraction and exploitation of lignin as the primary component and declare lignin the most valuable composite in lignocellulose. The Lignin-First approach entails a renewed assessment of lignocellulose processing focusing on the preservation of native, high-quality lignin properties. This new approach makes it more important than ever to understand the inter-polymeric connections in lignocellulose and target specific and selective cleavage of the anchoring points between lignin and cell wall polysaccharides. LCCs represents these essential anchoring points, and the precision-based enzyme-catalyzed hydrolysis of ester bonds is key to obtaining new and improved methods for exploiting lignocellulose in a future sustainable bioeconomy, where the aim is to avoid extensive and disruptive process conditions. Processing by glucuronoyl esterases offer a way to generate pure lignin-fractions, free from carbohydrates, as the enzymes can shave off any residual, covalently linked glucuronoxylan. The purity of lignin is an important quality parameter for efficient use in various applications. The fact that the ester-LCCs are chemically labile may be an important reason why these interconnection points have received relatively little attention before the discovery of glucuronoyl esterases.

## Diversity and CUPP classification

**Fig. 2:**
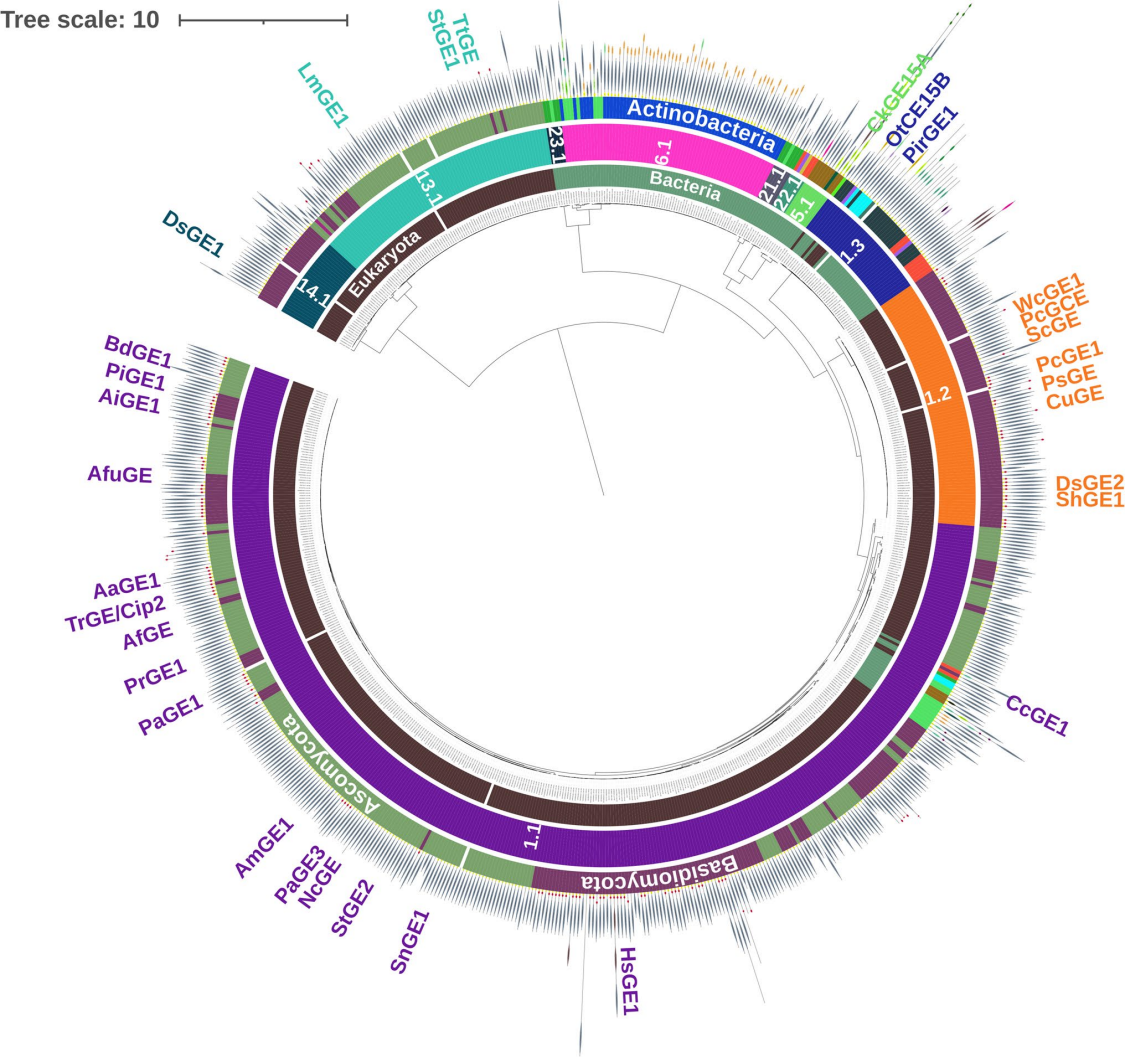
CUPP classification of CE15s—the “fungal branch.” The CUPP analysis overall divides all CE15s into either a major fungi-containing branch or a major bacteria-containing branch (only the fungi-containing branch is shown here). The fungi-containing branch does indeed also contain a considerable number of CE15s from bacteria. CE15s originating from eukaryotes (mainly fungi) and prokaryotes (bacteria) are distinguished on the inner band with brown and green, respectively. CUPP clustering in groups is marked by the middle band and labeled with individual CUPP-group numbers. Phylum is marked by the outer band. Characterized CE15s are indicated with names (e.g. CuGE for the glucuronoyl esterase from Cerrena unicolor). CE15 domains are shown as grey needle-like shapes on the outmost ring, while CBM1, CBM2, and GH10 domains are shown as red ellipses, orange ellipses, and brown needle-like shapes, respectively. The complete dendrogram and further information about other domains can be found by visiting the following link: CUPP dendrogram (https://shorturl.at/duKQV). Noticeably, the fungal GEs only carry CBM1 domains, while the bacterial GEs carry various and often multiple other CBM domains with CBM2 being the most common

GEs are widespread in nature, and a few central studies describe the occurrence in fungi and aim to cluster them based on diversity (Agger et al. 2017; Dilokpimol et al. 2018). Here, we have performed an expanded BLAST search on CAZys CE15 family and performed a similarity grouping based on CUPP (Conserved Unique Peptide Patterns) (Barrett and Lange 2019). The grouping by CUPP is based on identifying and organizing clades of enzymes each sharing similar motif groups; these motif groups are defined as peptide fragments comprising eight amino acids in length of which two are ambiguous. The CE15 proteins included in the analysis originate from CAZy.org or from a BLAST expansion (Camacho et al. 2009) using each experimentally characterized fungal CE15 protein as seed against the NCBI NCBI (National Center for Biotechnology Information) database. Each BLAST hit belonging to fungi were included in the further processing for domain identification by HMMER/dbCAN9 (Potter et al. 2018; Zhang et al. 2018). The representative domains were selected by CDHIT (threshold 90%) (Li and Godzik 2006) before they were subjected to CUPP clustering (Barrett and Lange 2019; Barrett et al. 2020) and displayed using iTol (Letunic and Bork 2021). In total, the analysis included 1398 sequences of which 599, 745, and 45 were from eukaryota, bacteria, and archaea, respectively (the remaining nine sequences were unknown). The eukaryotic GEs were almost entirely from fungi (596 of 599 sequences). The CUPP-analysis shows that the bacterial and fungal CE15s fall largely into two major branches (please visit CUPP dendrogram, Fig. 2). The fungal GEs are relatively more studied than bacterial GEs. The fungal GEs were then extracted into a new separate dendrogram denoted “the fungal branch”, Fig. 2. In this fungal branch dendrogram, the GEs cluster into five CUPP groups, namely CUPP 1.1, 1.2, 1.3, 13.1, and 14.1, and at least one fungal representative has been characterized in all of these groups (Fig. 2). Especially CUPP 1.1 and 1.2 are well characterized as several fungal CE15s belonging to these groups have been studied. On the other hand, although the interest in bacterial GEs has increased recently (Larsbrink and Lo Leggio 2023), bacterial GEs are still poorly explored. More recently, a new, easy-accessible screening methodology became commercially available via the Glucuronoyl Esterase Assay Kit (Megazymes, Ireland). The method is colorimetric, based on the eventual release of *p*-nitrophenol after a coupled enzyme reaction with a methyl-ester of a 4-*O*-methylated aldouronic acid as the initial substrate. Such assays are good examples of ways to increase the screening of new and potential GE-acting enzymes in a wider scale.

It is noted that the major branch of bacterial CEs contains a substantial number of bacterial GEs within the phylum Planctomycetes. These bacteria were originally classified as eukaryotes due to their unique characteristics such as compartmentalized-like cell plan and ability to divide by budding, and some studies propose a eukaryotic ancestry of the phylum, although the similarities between Planctomycetes and eukaryotes are presumably a consequence of convergent evolution (Wiegand et al. 2018). Interestingly, the major bacteria-containing branch contains one eukaryotic GE originating from the choanoflagellate *Salpingoeca rosetta* (CUPP group 16.1). Choanoflagellates are single-celled microeukaryotes found in marine and freshwater environments (Dayel et al. 2011). They have a cell morphology typically characterized by an ovoid cell body and a single apical flagellum, and feeds by phagocytosing bacteria (Dayel et al. 2011). It is tempting to speculate that *Salpingoeca rosetta* uses its glucuronoyl esterase to degrade yet unknown substrates from the marine environment.

## CBM domains

The main structural differentiator between the fungal GEs is the absence (78%) or presence (22%) of a single CBM1 domain (red ellipses, Fig. 2), which is attached either N-terminally or C-terminally to the catalytic CE15 domain through a linker-region. The fungal GEs cluster into the aforementioned five CUPP groups, where appended CBM1 domains can be found within all groups except CUPP 1.3. Appended CBM1 domains are most frequent in GEs from CUPP 1.2 (38%) and CUPP 1.1 (23%), while CBM1s are less common in CUPP 13.1 (9%) and CUPP 14.1 (4%). The N-terminal placement of the CBM1 domain is most typical in CUPP 1.1 (88.2%), 1.2 (83.3%), and 14.1 (100%), while all CBM1-containing sequences within CUPP 13.1 have their CBM1 domain positioned C-terminally to the catalytic domain. Noticeably, the fungal GEs only carry CBM1 domains, while the bacterial GEs may carry CBM domains from various different families and often carry multiple CBMs. Looking only on the major fungi-containing branch, the bacterial GEs herein may carry CBM domains of family 2, 3, 4, 5, 6, 9, 13, 22, 35, 57, and 60. The most common CBM domain found in bacterial GEs is CBM2 (Larsbrink and Lo Leggio 2023), and these GEs mainly originate from Actinomycetes. In terms of characterized fungal proteins, it appears that a large part of the diversity is covered by the current basic knowledge, whereas it is still poorly understood why only a quarter of the proteins carry a binding domain. The effect of the CBM1 domain on enzyme activity was inspected by (Mosbech et al. 2019) who investigated the kinetics of four fungal GEs either with a CBM1 domain (*Ps*GE, *Cu*GE) or without (*Afu*GE, *Tt*GE). Although no particular difference in activity was identified between the four variants, a truncated version of the glucuronoyl esterase from *Cerrena unicolor*, was shown to have a higher *K*_M_ and a lower *k*_cat_ compared to the full-length enzyme. Thus, the general role and the specificity of CBMs in relation to the function of glucuronoyl esterases is not understood.

The CUPP-analysis shows that some CE15s are bound to other catalytic domains such as CEs and GHs. For instance, the CE15s from the wood-degrading fungi *Mucidula mucida* and *Sphaerobolus stellatus* contain a single GH10 domain and two GH10 domains appended to their CE15 domain, respectively. This may indicate that these two enzyme activities can cooperate in degrading lignocellulose, which is supported by studies on the glucuronoyl esterase from the white-rot fungus *Cerrena unicolor* (Mosbech et al. 2018). As CE15 and GH10 domains are only found together in two of the fungal proteins it should not be ruled out that these occur together as a result of gene prediction errors. However, both GH domains and other CE families are commonly found together among the bacterial CE15s. Taken together, this bioinformatics based analysis of the CE15s indicates that this enzyme family indeed covers a number of distinctly differently composed enzymes, and suggests a new way to organize this enzyme family.

## Structure and function

**Fig. 3:**
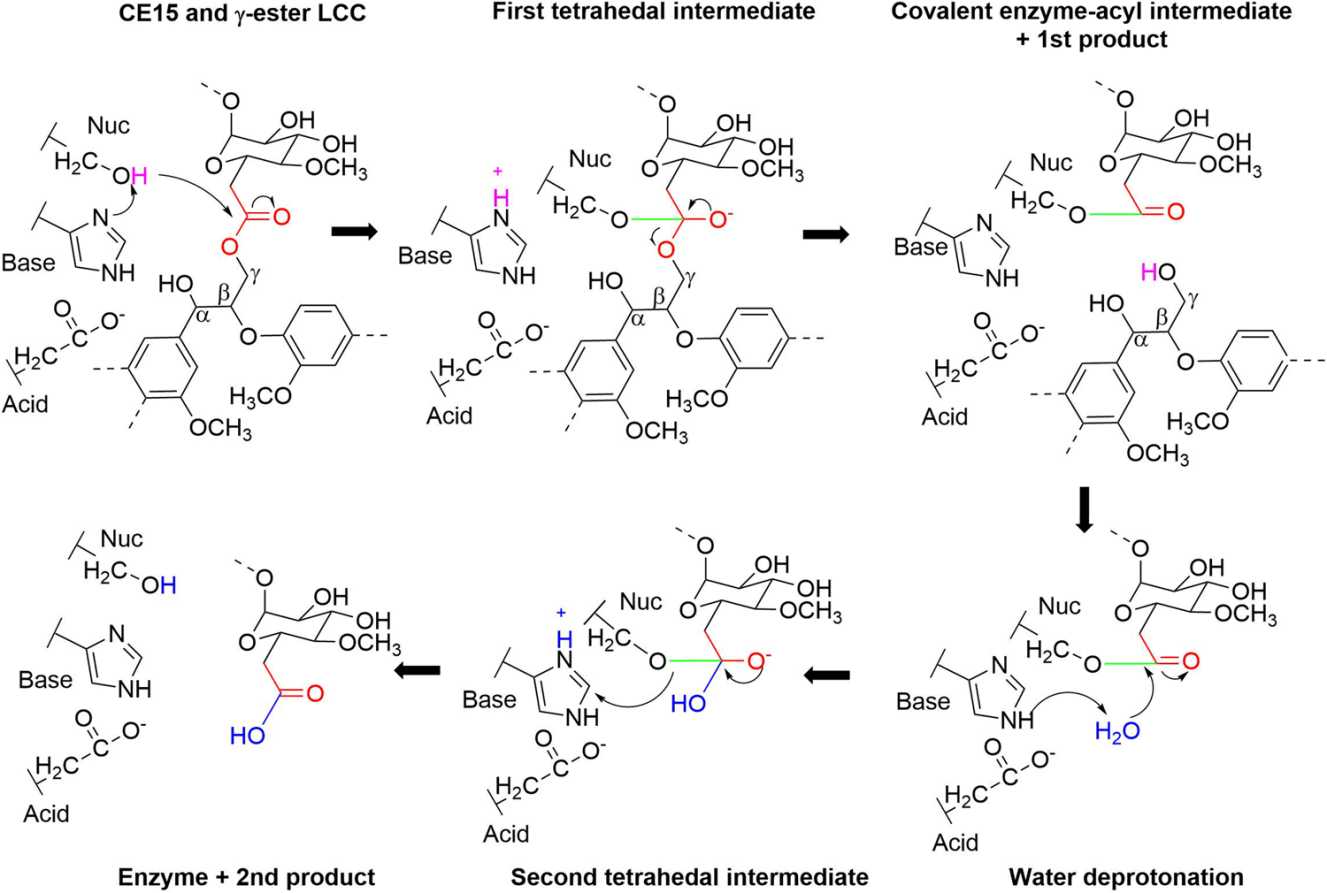
Catalytic mechanism of canonical fungal CE15 glucuronoyl esterases. The nucleophile (Ser) gets deprotonated by the base (His) and attacks the ester bond in the substrate. The role of the acid (Glu) is to stabilize the protonated histidine. The substrate is here drawn as γ-ester between the γ-positioned hydroxyl on lignin and the carboxylic group of α-1,2-linked 4-O-methyl-d-glucuronoyl substitution on xylan. The nucleophile forms a tetrahedral intermediate with the substrate, which leads to the formation of the enzyme-acyl intermediate and cleavage of the alcohol-part of the product when the base (His) donates the proton. In this case, the first product is an intact γ-hydroxyl on lignin. The alcohol-product leaves the active site and water enters and gets deprotonated by the base, and hereby becomes the second nucleophile, which exerts an attack on the enzyme-acyl bond and forms the second tetrahedral intermediate. The reaction ends when the serine gets protonated by histidine, and the carboxylic acid-product leaves the active site. In this case, the carboxylic acid-product is an aldouronic acid with an unspecified degree of polymerization on the xylan-backbone

Glucuronoyl esterases belong to the superfamily of α/β-hydrolases and possess the classical serine-based catalytic triad of Ser-His-Glu in their active site. They all share a common consensus sequence consisting of GC**S**RXG, surrounding the conserved catalytic serine (Topakas et al. 2010). Structural studies have clearly established the catalytic residues, and the mechanism of catalysis follows that of the canonical serine esterases (Fig. 3) but is unique for glucuronoyl esterases in the sense that large substrate molecules accommodate into the active site (Pokkuluri et al. 2011; Charavgi et al. 2013). In particular, large and bulky alcohols bind stronger to the enzyme compared to small methyl esters (Ďuranová et al. 2009a; d’Errico et al. 2015), and there is a strong recognition for the 4-*O*-methylation of the glucuronoyl moiety (Ernst et al. 2020), which largely defines the specificity of these enzymes. In the presence of another hydroxyl group than water, these enzymes might most likely be prone to catalyze the transfer of the glucuronoyl to another acceptor, leading to transesterification rather than hydrolysis (Fig. 3). However, such events have not been reported experimentally yet.

The first CE15 structure in complex with a ligand; the soluble substrate analog 4-*O*-methyl-d-glucuronoyl methyl ester, confirmed the oxy-anion-hole that stabilizes the tetrahedral intermediate (Fig. 3) as formed by the backbone N-atom and the guanidinium-N of a central asparagine residue in the active site. Recent QM/MM studies of a bacterial GE, *Ot*CE15A indicate that the rate-limiting step in catalysis is the deacylation of the enzyme-acyl intermediate and that the arginine participating in the oxy-anion-hole formation is a key player in the final hydrolytic steps (Fig. 3), despite it is not recognized as one of the catalytic residues (Zong et al. 2022).

The role of the aspargine was also observed in the first structure from the basidiomycete *Cerrena unicolor* (Ernst et al. 2020). This latter study also reports the first SAXS-structural characterization of a GE (*Cu*GE) in full length and indicates that the catalytic domain has a spatial elongation of approx. 60 Å, and the CBM is approx. 28 Å. The two domains are connected by a short linker-region, which yields a protein of around 110 Å in total. The globular structure of *Cu*GE informs about an elongated protein that potentially spans a contact range of up to 10 xylosyl-residues in the xylan-backbone.

In addition to the crystal structures of fungal GEs, a portfolio of crystal structures of bacterial counterparts have also been reported (De Santi et al. 2017; Arnling Bååth et al. 2018; Baath et al. 2019; Mazurkewich et al. 2019). Among the more prominent findings are the large inserts close to the active site that the fungal enzymes appear to be missing, and an alternative position for the catalytic acid (Baath et al. 2019). It was also in the bacterial enzymes that an alternative catalytic acid, aspartate was first observed (De Santi et al. 2017) and the structural analyses also explained the higher substrate promiscuity among the bacterial proteins compared to the fungal ones (Arnling Bååth et al. 2018). Most recently, a bacterial CE15 from a multi-domain protein from *Caldicellulosiruptor kristjansonii* was also added to the collection of crystal structures (Krska et al. 2021), and this enzyme specializes by being one domain out of a large complex consisting of seven domains (one xylanase and five CBMs in addition to the CE15).

Regarding the catalytic acid; due to the differences in position and type of acid that has been observed among both fungal and bacterial GEs we have proposed a differentiation into two groups, CE15A or CE15B proteins (Ernst et al. 2020). A-variants have the catalytic aspartate positioned after β-strand 8 and the B-variants have the catalytic glutamate positioned after β-strand 7.

## Possible lignin interactions

One of the most urgent questions is how these enzymes accommodate the lignin-part of the substrate. It is a difficult question to answer unequivocally because of the high heterogeneity and insolubility of lignin, and even simple model compounds are difficult to produce. So far, there has been no documented successful attempts of characterizing an intact LCC-enzyme complex or even a CE15 in complex with a substrate analog.

**Fig. 4:**
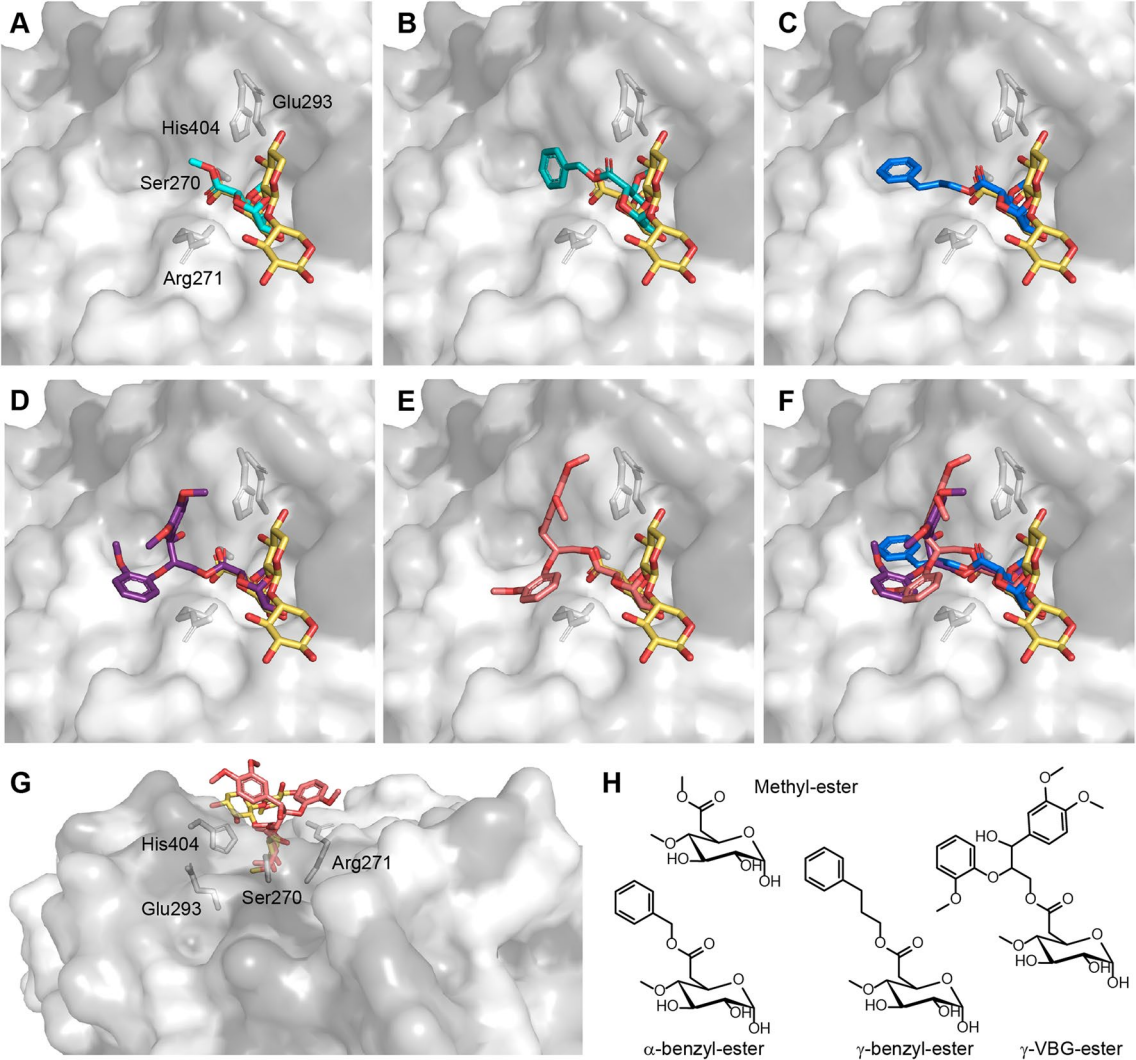
Docking experiments^#^ of various potential model substrates in the active site of *Cu *GE (PDB 6RU1). Each panel (**A**–**F**) contains examples of best fits with each of the model-ligands (panel **H**). Coloring of the enzyme surface is done according to the Normalized consensus hydrophobicity scale (the Eisenberg scale), where drak grey represents hydrophobic residues and white represents hydrophilic. **A** Docking of the methyl-ester of α-d-glucuronic acid. Important catalytic residues (Ser270, Glu293, and His404) are highlighted and labeled together with Arg271, which forms the oxyanion hole. All active site residues are highlighted in all panels (**A**–**G**), but they are only labeled in **A** and **G**. Note that the nucleophilic serine is placed behind the ester-oxygen. **B** Docking of an α-benzyl-ester of α-4-O-methyl-d-glucuronic acid. **C** Docking of a γ-benzyl-ester of α-4-O-methyl-d-glucuronic acid. **D** One example of docking of a veratrylglycerol-β-guaiacyl ether γ-ester linked to α-4-O-methyl-d-glucuronic acid (γ-VBG-ester). **E** Another example of docking of the γ-VBG-ester showing a slightly different orientation of the ring-structures. **F** Stacking of **C**, **D**, and **E** in the same view. **G** Side view of **E** to illustrate the spatial orientation of the aryl-group. Note that only the glucuronoyl is docking into the active site, whereas the majority of the substrate molecule is hovering over the flat surface of the protein. Active site residues are labeled for clarification. **H** Chemical structures of the four different ligands. ^#^AutoDock Vina was run through the PyMOL plugin DockingPie 1.0.1 (Rosignoli and Paiardini 2022). In DockingPie, the Vina function was chosen where CuGE was set to the receptor, either one of the four esters in Fig. 4H were set to the ligand, and the docking cavity was a grid limited to the active site in the crystal structure. Exhaustiveness was always set to 40, Energy Range to 3 and Poses to 20. The in silico dockings were evaluated by their proximity to the glucuronoyl moiety of the crystal structure

For the purpose of making an in silico assessment of the putative lignin-binding site in the glucuronoyl esterases, we performed molecular docking simulations with *Cu*GE (PDB: 6RU1) and relatively simple substrate analogs. With this protein structure (PDB: 6RU1), *Cu*GE exists in complex with an aldotetrauronic acid. This complex served as a starting point for molecular docking where the glucuronoyl moiety functioned as a guide for the new ester-ligands. The goal was to assess the possible orientations and interactions between the proteins and the aryl-half of the ester-linkage to an α-4-*O*-methyl-d-glucuronic acid (Fig. 4). AutoDock Vina (Trott and Olson 2010; Eberhardt et al. 2021) was used for in silico docking of the methyl-ester and three different aryl-esters that were chosen to represent either a gamma-ester, an alpha-ester or a lignin-like gamma-linked ester (Fig. 4H).

The molecular docking of the simplest methyl-ester (Fig. 4A) shows that it is possible to guide the docking so the glucuronoyl moiety falls almost perfectly on top of the glucuronoyl from the original ligand in the complex, and as expected the ester bond positions closely to the catalytic serine. The gamma-benzyl-ester (Fig. 4C) behaves similar to the methyl-ester in terms of glucuronoyl overlap, which results in the benzyl elongating parallel along the surface of the protein away from the aldouronic acid but without any direct interactions. On the contrary, it was not possible to obtain perfect alignment between the glucuronoyl of the alpha-benzyl-ester and the glucuronoyl of the aldouronic acid (Fig. 4B), suggesting unfavorable configuration of the relatively short aliphatic chain of the alpha-benzyl compared to the gamma-benzyl. As the complexity of the docking ligand increases, the number of possible configurations also increases. However, the best alignments of the glucuronoyl moiety to the aldouronic acid with the gamma-veratrylglycerol-β-guaiacyl ether-ester (gamma-VBG-ester) (Fig. 4D and E) all imply that the aryl-group will follow the same orientation as the gamma-benzyl (Fig. 4F) even though with larger flexibility in terms of distance to the protein surface and rotation around the chiral centers. The gamma-VBG-ester has two chiral centers (around the α- and β-carbon), and overall, the different enantiomers did not show significant differences in propensity for docking, albeit the S-R enantiomer may position itself slightly closer to the enzyme surface (data not shown). The shortest distance in these dockings between the VBG-ligand and the protein appear between Arg271 and the ring-carbons of the ligand (Fig. 4E). The distance is estimated to be between 3.6 and 3.8 Å, and therefore too long for hydrogen bonding, but potentially enough for Van der Waals forces. Changes in pH within a relevant range are not expected to affect the interactions to this Arg271 due to its high pK_a_. The overall impression is that indeed the protein can accommodate the aryl-moiety (Fig. 4G) even though merely simulated here with a lignin-fragment. *Cu*GE belongs to the major CE15-B group (Ernst et al. 2020), and these proteins lack two specific structural inserts between β-sheet no. 4 and 5 compared to the major CE15-A group of proteins. The latter CE15-A group includes many of the bacterial GEs. This lack of inserts in the B-group results in a flat and open surface on the protein around the active site, whereas those belonging to the CE15-A group present a bulky topology on the putative lignin-binding site. How this bulky difference in surface topology affects the lignin binding is still unknown. It is therefore central to emphasize the importance of experimental verification of these molecular docking simulations, albeit it is tempting to speculate that the enzyme itself has limited or no direct interactions with the lignin polymer in the natural substrate, and that the lignin-part of the complex is oriented orthogonal to the xylan backbone. Both previous observations that GEs release long aldouronic acid-based oligosaccharides (Mosbech et al. 2018) and these docking models indicate that GE activity is not dependent on preliminary xylanase or α-glucuronidase activity.

## Expression during fungal growth

GEs constitute part of the enzymatic arsenal used by fungi to overcome the recalcitrant nature of lignocellulose. Like other CAZymes, the expression of these enzymes is highly linked with the nature of the cultivation substrate. For instance, when cultured in sugarcane bagasse as substrate, the CE15 of *Gloeophyllum trabeum* was more abundantly expressed than when cultivated only in glucose (Valadares et al. 2019). Moreover, GEs are expected to act in synergy with other carbohydrate-active enzymes (CAZymes), such as xylanases and α-glucuronidases, revealing potentially high-impact synergistic effects as previously demonstrated in vitro (Mosbech et al. 2018; Raji et al. 2021). The fungal adaptation to a given growth medium is seemingly caused by differential expression of certain genes rather than expression of unique genes (Janusz et al. 2018). Although there are currently no studies focusing on GE expression in wild type strains, these enzymes have been identified in the set of secreted enzymes as evaluated by secretome and transcriptomic analyses of fungi grown on lignocellulose (Alfaro et al. 2014; Hori et al. 2020; Miyauchi et al. 2020). For instance, the composition of *Ceriporiopsis subvermispora* secretome was studied over time when the fungus was grown on aspen wood, identifying different expression patterns for two CE15 proteins (Hori et al. 2014). At days 5 and 7, the first CE15 (ID21396) was listed among the ten most abundant proteins in the secretome. The protein was already detected at day 3 and its expression kept rising up until the last evaluated point. The second CE15 (ID118322) maintained similar levels at days 3 and 5, but was not found at day 7. Similarly, other studies also showed that GEs were expressed during the early days of cultivation (Bengtsson et al. 2016; Miyauchi et al. 2017; Hori et al. 2020; Daou et al. 2021) being the earliest detected CE at two days of cultivation (Presley et al. 2018). Along these lines, one can infer that GE constitute part of the primary group of secreted enzymes, helping to loosen up carbohydrate fibers which end up giving more access to other depolymerizing enzymes and non-catalytic proteins that constitute the system for breakdown of lignocellulose. Contrastingly, when the white-rot fungus *Phlebia radiate* was grown on spruce wood for six weeks, two CE15 enzymes were detected with the first (minus.g10669) peaking at growth day 14 and the second (minus.g1785) increasing throughout the experiment, indicating that GEs may not only exhibit a role in the initial phase of lignocellulose degradation (Kuuskeri et al. 2016). Hitherto, the majority of secretome and transcriptomic studies on fungi growing on woods have begun sampling after the fungus has grown for several days on its substrate, while studies focusing on enzymes secreted during the initial growth or first days of growth are lacking.

Furthermore, it is interesting to consider the production and secretion of GEs from an ecological point of view because of the inter-microbial synergy between biomass-degrading microorganisms. It is reasonable to assume that each secretome composition within a microbial community will vary not only in accordance to the colonized substrate, but also regarding the biodiversity of the community. In that sense, GE-producing microorganisms could have co-evolved to be part of the primary colonizers to attack lignocellulolytic substrates being followed by other members of the community that harbor genes to contribute with other sorts of CAZymes. Hence, the interactions between microorganisms in terms of contribution to the enzymatic pool are critical to the survival of the community and an improved understanding of the diversity of contribution of enzymes provide insight into the natural complexity of the ecological succession and chemical biology of lignocellulose deconstruction (Bomble et al. 2017).

## Closing remarks

The current knowledge about glucuronoyl esterases and their activity toward various substrates and substrate analogs provides detailed information on how the enzymes bind and interact with the carbohydrate moiety of the LCCs. The recent reclassification of these enzymes as (4-*O*-methyl)-d-glucuronate-lignin ester hydrolase (EC 3.1.1.117) is refreshing, but it is noteworthy that descriptions of how the enzymes accommodate lignin are incomplete. In this review we addressed the question of possible interactions and orientation of the lignin-moiety of the substrate in the active site by modeling a plausible sub-fragment of the ester-LCC-complex into the known structure of *Cu*GE. The results indicate no direct interactions between the active site of the enzyme and the lignin-like structure, and that the lignin structure is oriented away from the surface of the protein in a perpendicular direction compared to the carbohydrate backbone. Finally, the docking experiments indicate that an elongated aliphatic chain between the phenyl and the ester linkage, corresponding to a γ-ester is favorable for correct positioning of the glucuronoyl compared to the corresponding α-ester. Fungal variants of the GEs inevitably show strong recognition of the 4-*O*-methylated glucuronoyls and non-alignment based clustering using CUPP revealed that most fungal GEs cluster together in a “fungal branch.” Even though gaps exist in the characterization of proteins in the fungal branch, most diversity seem to be covered in terms of establishing the enzyme activity, with the exception of a few bacteria-dominated CUPP groups in that same branch. The major diversity and missing links for the fungal proteins appear in the significance and role of their domain organization with binding modules, and potentially in any specificity toward lignin. The next major accomplishment in GE research is inevitably linked to application and process strategies, where we foresee that the potential of these enzymes will be realized to benefit the sustainable exploitation of lignocellulose.

## Supplementary Information

Below is the link to the electronic supplementary material.

Supplementary file 1 (XLSX 98 KB)

## Data Availability

All data used to generate the reported results are based on publicly available databases; CAZy, NCBI and PDB. The link to the CUPP dendogram includes all data related to the CUPP analysis.

## Declarations

This article does not contain any studies with human participants or animals.
